# Nanotube abundance from non-negative matrix factorization of Raman spectra as an example of chemical purity from open source machine learning

**DOI:** 10.1038/s41598-022-15359-4

**Published:** 2022-07-08

**Authors:** Elijah Flores, Jianying Ouyang, François Lapointe, Paul Finnie

**Affiliations:** 1 National Research Council Canada, 1200 Montreal Road, Ottawa, ON K1A 0R6 Canada; 2 University of Waterloo, 200 University Avenue West, Waterloo, ON N2L 3G1 Canada

**Keywords:** Carbon nanotubes and fullerenes, Analytical chemistry, Carbon nanotubes and fullerenes

## Abstract

The chemical purity of materials is important for semiconductors, including the carbon nanotube material system, which is emerging in semiconductor applications. One approach to get statistically meaningful abundances and/or concentrations is to measure a large number of small samples. Automated multivariate classification algorithms can be used to draw conclusions from such large data sets. Here, we use spatially-mapped Raman spectra of mixtures of chirality-sorted single walled carbon nanotubes dispersed sparsely on flat silicon/silicon oxide substrates. We use non-negative matrix factorization (NMF) decomposition in scikit-learn, an open-source, python language “machine learning” package, to extract spectral components and derive weighting factors. We extract the abundance of minority species (7,5) nanotubes in mixtures by testing both synthetic data, and real samples prepared by dilution. We show how noise limits the purity level that can be evaluated. We determine real situations where this approach works well, and identify situations where it fails.

## Introduction

The determination of chemical purity is a persistent need and techniques are continually being developed. In the nanocarbon field, the purity of single walled carbon nanotube (SWCNT) dispersions is important because the (opto)electronic properties of bulk materials can change drastically due to the presence of unintended minority species. Chirality-purified dispersions of SWCNTs are increasingly available [i.e. (*n*,*m*) pure samples where *n* and *m* are indices identifying the chiral structure]. This is useful because (*n*,*m*)-pure SWCNTs have clear, well-defined electronic structures (e.g. with a fixed semiconductor band gap) and are therefore well suited to semiconductor and optical applications. In general, however, these materials have some level of “contamination” by other unwanted (*n*,*m*) and it is important to know how pure these materials actually are.

Raman spectroscopy is analytically powerful in this situation. It is a well-established tool for chemical analysis in general^1^ including quantitative chemical analysis^2^. It is especially useful for characterizing SWNCTs, distinguishing their types and chiralities, and is equally useful for other types of nanocarbons^3,4^. It enables the assessment of high purity nanotube materials required for semiconductor devices^4–6^. Micro-Raman spectroscopy instruments can scan hundreds or thousands of points on a sample and produce a large number of spectra. These large datasets are complex and it can be a challenging task to evaluate them. Fortunately, the analysis of such large datasets is facilitated and automated by multivariate “machine learning” algorithms.

There are various ways of determining SWCNT chirality distributions, and many of these have been reviewed recently^4,7,8^. Chirality can be determined by transmission electron microscopy or by optical spectroscopy. Optical spectroscopic methods are effective and relatively straightforward to scale to large amounts of material and large numbers of samples. For complex mixtures, photoluminescence excitation (PLE) mapping and Raman spectroscopic excitation mapping are two important methods. PLE may be the most important approach, but it is applicable only to the semiconducting species, and is very sensitive to sample preparation. Raman spectroscopy is applicable to almost any type of sample, solid or liquid and can be used for essentially all chiralities, if enough wavelengths are used. Raman spectroscopy has been important from the beginning and it is receiving renewed scrutiny as a quantitative analytical tool. The question now is not whether it is possible to quantify distributions of nanotube chiralities via Raman spectroscopy, but rather how representative, precise and accurate this spectroscopic technique can be^9–11^. It is a field where machine learning methods—like the decomposition used here—have the potential to be important by automating the analysis and drawing statistically meaningful conclusions.

Recently, open source machine learning-related software has become widely available. The use of open source software has great promise to make these methods more metrological. This is because such software is designed to be shared, and so is verifiable by essentially any interested party. The python computer language has many shared libraries which can simplify the practical implementation of sophisticated data science algorithms. A variety of such algorithms can be—and are—used to analyze Raman spectral mapping data. Especially, it is often useful to reduce large data sets to their main features, which may then be used for categorization, qualitative analysis and even quantitative analysis. Principal Component Analysis (PCA)^2^ is probably one of the most popular such multivariate algorithms, but the physical meaning of the components can be difficult to interpret as they relate only to variability and can take on any value positive or negative.

A less popular, but very promising and powerful algorithm for reducing and analyzing sets of Raman data is Non-Negative Matrix Factorization (NMF)^12^. Although NMF occupies a relatively smaller niche in the analytical toolkit at present, it has already been used to evaluate Raman spectroscopic data, especially for biological materials. It has recently been applied to plant cell wall analysis^13^, for cancer cell classification^14–16^, to analyze the structure of biofilms^17^, and to determine distributions of skin constituents^18^. It has also been used for the analysis of surface enhanced Raman spectroscopy (SERS) of food contamination^19^, and as a step in the evaluation of lipid content from coherent anti-stokes Raman spectroscopy (CARS) data^20^. For nanocarbons, it has been used to isolate the pure spectrum of graphene^21^.

Unlike PCA and many other multivariate algorithms, NMF decomposes data into positive value components only. Since, physically, Raman scattering generates only positive definite spectral features, the NMF-derived components can be straightforwardly relatable to the Raman scattering data. Here, the data are sets of simple spectra, essentially sets of vectors, and the extracted NMF components are vectors, which are then interpretable themselves as “effective” Raman spectra. The ideal is that the NMF components extracted from samples of mixtures of chemicals are identifiable directly as spectra from the individual compounds that make up the mixture. That is, for a mixture of (*n*,*m*) SWCNTs each with a different Raman spectrum, the ideal decomposition would be for each NMF component to be the spectrum of an individual (*n*,*m*) SWCNT species.

In the python computer language, the open source ”scikit-learn” machine learning package^22^ includes NMF decomposition. In this work, we apply scikit-learn’s implementation of NMF to decompose spatial Raman spectral mapping datasets and evaluate the purity of highly purified, “single chirality” SWCNT samples. We find it is a practical way to extract quantitative relative abundance information because the components do often correspond to spectra which can be identified with SWCNT constituents, and the weights and component outputs can then be used to determine the abundance of that SWCNT constituent. We examine how the algorithm performs on simulated data, with the advantage that we know everything about the synthetic samples, and we apply it also to evaluate the purity of real prepared mixtures of high quality, pure (*7*,*5*) and (*6*,*5*) chirality nanotubes deposited on silicon substrates. We test the limits of purity assessment in this way as a function of the quality of the spectral data.

## Results

**Figure 1:**
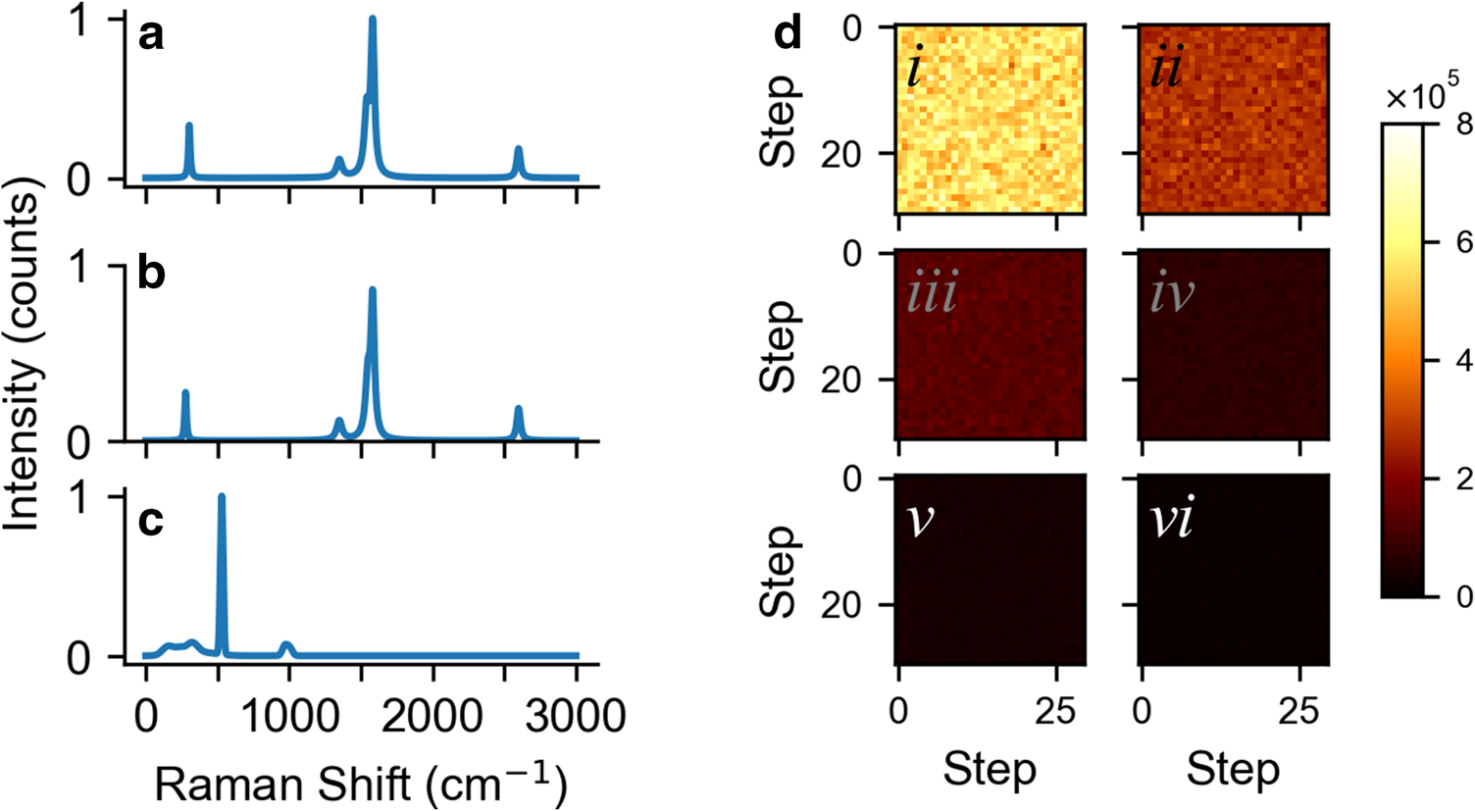
Synthetic data. Simulated Raman spectrum of (**a**) a (6,5) nanotube, (**b**) a (7,5) carbon nanotube, (**c**) the silicon substrate. (**d**) Spatial maps of the radial breathing mode intensity for a series of simulated (7,5) samples with decreasing concentrations corresponding to (*i*–*vi*) 2×, 4×, 8×, 16×, 32×, and 64× dilutions respectively. The color indicates the number of tubes present in each pixel.

To test the NMF decomposition on a fully known data set, we simulated the Raman spectrum of (*6*,*5*) and (*7*,*5*) nanotubes (Fig. 1a,b). Details of the simulation in the “Methods” section. Briefly, we simulated a Radial Breathing Mode (RBM), a D band, overlapping G^−^ and G^+^ bands, and a 2D band^3^, but did not include any of the many other, usually weaker, SWCNT bands. The Raman shifts were chosen to be realistic, though the band intensities were chosen arbitrarily. They are, however, more-or-less typical of SWCNT Raman spectra in general. For the substrate (Fig. 1c) we simulated first (520 cm^−1^) and second order (~1000 cm^−1^) silicon peaks, and also the lower frequency, broad structures seen in the ~ 100 cm^−1^ to ~ 300 cm^−1^ range on silicon dioxide/silicon samples. The simulated spectra are simplified: every nanotube is assumed to have the exact same spectrum, including intensities. We then simulated “synthetic samples” where these nanotubes were distributed randomly on 30 × 30 grids, at different simulated dilution factors for the (*7*,*5*) nanotube. The number of nanotubes in each pixel was allowed to be a non-integer and was represented by a floating point number. We show spatial maps of the number of nanotubes for these synthetic scans (Fig. 1d). As the dilution factor increases, the average intensity of the pixels on the map decreases.

To be definite about the analysis, for the NMF decomposition, each spectrum can be considered a vector ***s*** (i.e. a vector of intensities recorded at a series of wavenumbers)*. *The set of spectra from all spatial points on the map is the matrix ***S*** = {***s***_1_,***s***_2_…***s***_*N*_}¸where *N* is the number of spatial points sampled. Here *N* = 30 × 30 = 900. The NMF algorithm solves for a decomposition of this matrix into two matrices ***W*** and ***C.*** We use a (small) set of *M* of components ***C*** = {c_1_,***c***_2_…***c***_*M*_} for all spatial points, where the component vectors ***c ***have the same dimensions as the spectral vectors**.** Here ***W*** is the matrix of weights that we multiply the set of components by to generate the best possible representation of the spectra at each data point. There is a separate weight for each of the *N* pixels in the spatial map, and each of the *M* factors**.** The solving algorithm minimizes the distance between ***S*** and ***WC.*** This distance can be defined in various ways, and we used the most standard “Frobenius norm”, which is simply the Euclidean distance (squared) between the two.

Physically, we can interpret the components ***C*** as a set of Raman spectra. Ideally, each of these components would correspond to a different chemical constituent making up the sample, in this case different species of nanotube, or perhaps the substrate’s background spectrum. For example, a sample that could be represented by two components ***C*** = {***c***_1_, ***c***_**2**_} would lead to ***w***_1_**c**_1_ + ***w***_2_**c**_2_ as the decomposition representing ***S***, with the weights ***w***_1_ and ***w***_2_ describing how much of the two components are present in the spectra at each measured pixel. Ideally, the components ***c***_1_, ***c***_**2**_ would be identifiable as the Raman spectra of the chemical constituents the sample (i.e. ‘compound 1’ and ‘compound 2’).

We want to relate the output components to the amount of each chemical constituent. Even if NMF matches the output components with identifiable spectra of constituents, there is a well-recognized and inherent ambiguity in the NMF decomposition. It is always possible to multiply the components ***C*** by a constant, and divide ***W*** by the same constant, and get an equally good solution. This is true for each component separately (along with the subset of weights corresponding to that factor). That is, the components can be normalized essentially arbitrarily with respect to the Raman signal that would represent a given quantity of the constituent.

So, to determine how much of any constituent is present, it is necessary to consider both the weights and the scale of the components. If we are able to identify the components ***c***_*i*_ with the Raman spectrum for each compound (“*i*”), this means the factor is proportional to the Raman spectrum, and does not represent the spectrum in absolute terms. This is not necessarily too consequential: Experimentally, Raman spectra are rarely calibrated in absolute terms, so usually the measured spectral intensity is a related to the absolute concentrations by a constant factor anyway. So, if a species is represented by a component ***c***_*i*_ its amount in any pixel is represented by the weight for that pixel times the factor. Or for the entire map, it is the sum of all the weights times that factor. The point is, it is a normalization factor for the component, times its weight which represents the abundance, not the weight alone.

**Figure 2:**
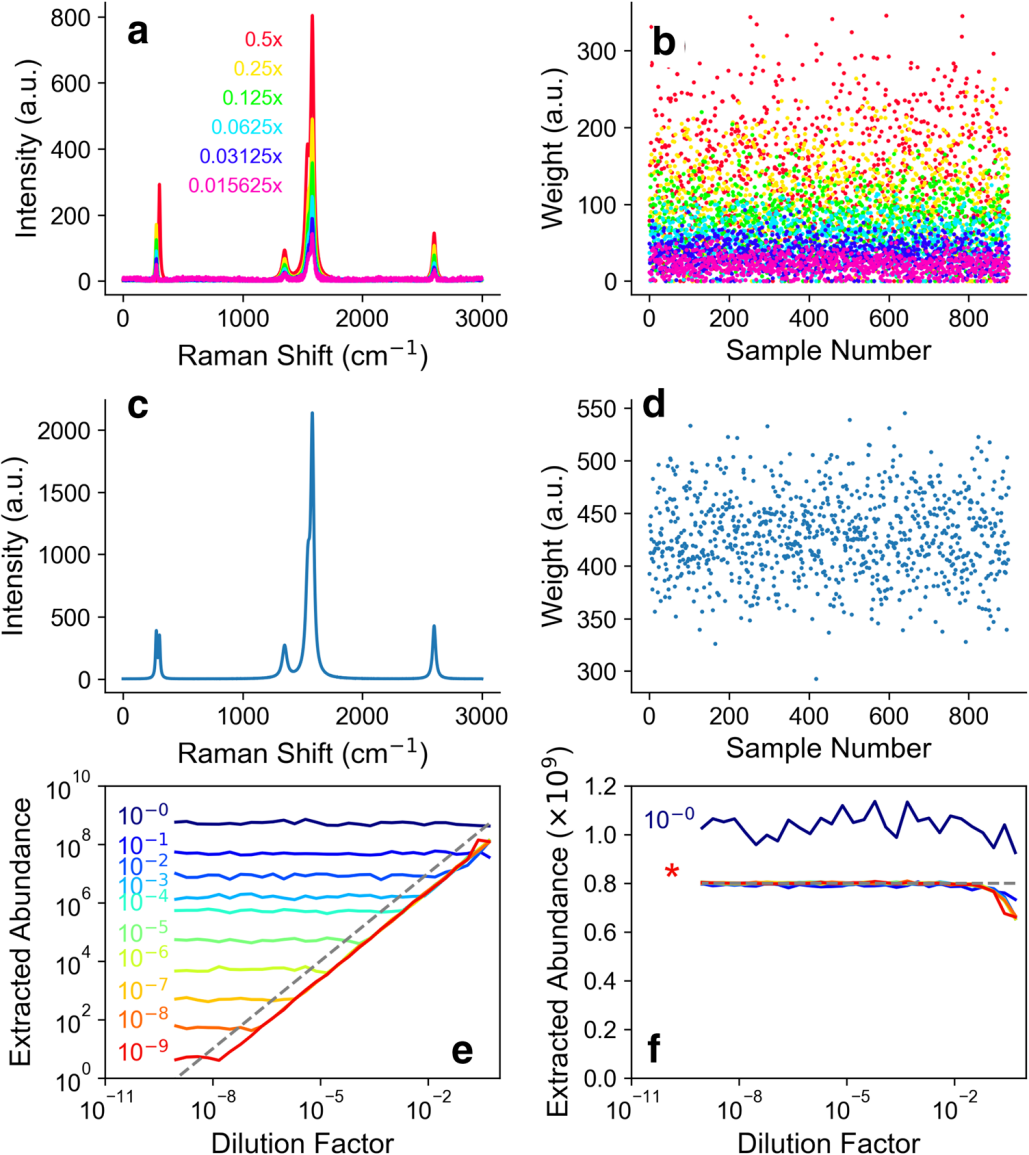
Analysis of synthetic data. (**a**) Extracted factor identified with minority (7,5) species and (**b**) the corresponding weights. Colors in (**a,b**) represent different dilution factors. (**c**) An example of the extracted factor identified with the majority (6,5) species (**d**) the corresponding weights. (**e**) Extracted abundance of the minority (7,5) species at different noise levels. The dashed line represents the true abundance. (**f**) Extracted abundance of the (6,5) species at different noise levels on a linear *y*-axis scale. In (**e,f**) colors represent different noise levels (see main text).In (**e,f**) colors represent different noise levels (see main text).

Figure 2 shows the result of NMF decomposition analysis of the above data with scikit-learn. Figure 2a shows the factors that are extracted, with different colors for different concentrations. Here, the particular case of noise level (the inverse of the signal-to-noise) of 10^–3^ is shown (see “Methods” section for details). That noise level is on par with the experimental noise level in real experimental data. We have not constrained the intensities of the components, and we can see that different concentrations give rise to very similar components, and these can all be identified as different multiples of our synthetic (7,5) species spectra. Figure 2b shows the corresponding weights. The weights vary pixel to pixel, but decrease, on average, in order of concentration. The amplitude of the components in (a) times the weights for a given pixel in (b) represents the amount (*7*,*5*) nanotube material in a given pixel.

Figure 2c shows the factor corresponding to the (*6*,*5*) majority species, and Fig. 2d shows the corresponding weights for each pixel. Since the majority species concentration is unchanged, the components and the distribution of weights changes little for different concentrations, and so one component and set of weights is shown, as an example, corresponding to the case of 1000× dilution only.

In reality, the Raman cross-section is different for each (*n*,*m*). Being resonant, the cross-section depends strongly on the choice of laser wavelength. Each Raman band has its own cross-section and—less commonly understood—not only does each (*n*,*m*) have its own laser wavelength dependence, but each Raman band for any given (*n*,*m*) has a somewhat different laser wavelength dependence. The (6,5) and (7,5) come from different “mod” [sets of (*n*,*m*) where 2*n* + *m* modulo 3 is a different integer]. Because of this, despite having similar diameters, their cross-sections differ significantly^9^. To obtain true relative abundances in real world measurements, the relative difference in cross-sections must be considered. However, we do not correct for this here.

Figure 2e summarizes the ability of the algorithm to determine the minority species abundance with synthetic spectral “data” of various qualities, created by simulating different signal-to-noise ratios (S/N). We use a harsh definition of signal-to-noise, with the signal defined as the intensity of the strongest G band signal in the set, and the noise defined as the root-mean-squared (rms) fluctuation. This is harsh in that it tends to overestimate the S/N since the average G band intensity is lower than the strongest G band intensity, and since both species have a similar G band. The actual information content which distinguishes them might only come from other bands, such as the RBM band, which is almost an order of magnitude weaker than the G band. In other words, if two species produce the same spectral feature, then although it is a signal of both species, it is not a signal that distinguishes these species.

In Fig. 2e, the dotted line represents the true abundance in the synthetic data. The colored lines represent the extracted abundance at different signal-to-noise levels, set to range over 9 orders of magnitude. As the S/N increases (i.e. the noise level at the left drops) it becomes possible to measure abundance at lower and lower levels. The number gives the S/N in units of the maximum G band (i.e. with the maximum G band set to 1). For example, with a noise level of 10^–4^ corresponding to S/N ~ 10^4^ for the G band, the abundance is extracted down to a floor of about 10^6^ SWCNTs. We have simulated 10^9^ SWCNTs, so this means measurement of a chiral purity better than one part per thousand is achievable at this level of S/N. Higher purity assessment requires better S/N.

Except in the case of extremely high noise levels, in Fig. 2e the extracted abundance follows the true abundance down to a level where the curve flattens out to horizontal due to the noise level. There is a small shift of the extracted abundance from the true abundance. This is likely explained by the mis-assignment of some of the weight to the factor that we identify as the (*6*,*5*) minority species to other components. Figure 2e can be used as a guide to identify how good the signal-to-noise ratio must be to measure a given purity level.

Figure 2f shows the extracted majority (*6*,*5*) species abundance. For the top curve, the noise is so high (S/N ~ 1) that the extracted abundance is quite far off. All the other noise levels (labelled by the * in the figure) overlap and represent the true majority abundance well, except at the lowest dilution factor, where the minority species concentration is so high it is only barely a “minority” species and the algorithm does not separate (*6*,*5*) and (*7*,*5*) well. Supplementary Material Figure S1 shows the same data as Fig. 2e,f in terms of concentration of minority species. This graph can be summarized by a simple rule of thumb that the minority species concentration is measurable down to the order of magnitude of the noise level in the spectra (inverse of the S/N).

After exploring the performance of scikit-learn’s NMF algorithm on synthetic data, we created real samples of purified, predominantly (*6*,*5*) and (*7*,*5*) nanotubes. These species were polymer-wrapped and suspended in toluene mixtures. The dispersions were then deposited by a soaking process on silicon dioxide on silicon (SiO_2_:Si) substrates. A series of dilutions of the “minority” (*7*,*5*) species were prepared in “majority” (*6*,5) dispersions. These mixed dispersions were also deposited by a soaking process on the same substrates (see details in “Methods” section).

**Figure 3:**
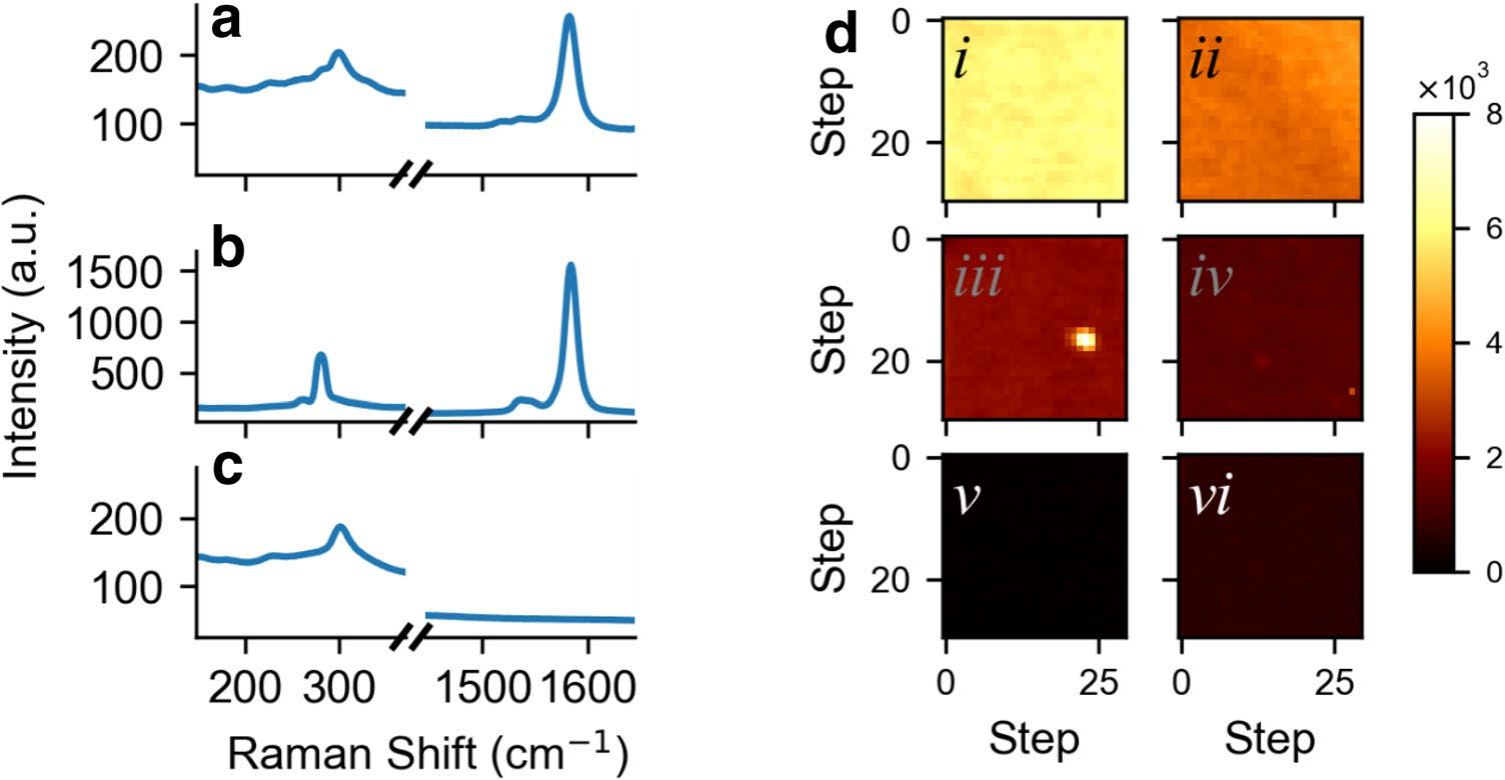
Real data. Raman spectrum of (**a**) a (6,5) nanotube sample, (**b**) a (7,5) carbon nanotube sample, (**c**) the silicon substrate. (**d**) Spatial maps of the Radial Breathing Mode intensity for a decreasing concentrations corresponding to (**a–f**) 2×, 4×, 8×, 16×, 32×, and 64× dilutions respectively. The color indicates the intensity of the RBM in each pixel. Grids are 30 pixels by 30 pixels with each pixel separated by 10 µm in the *x* and *y* directions.

Figure 3 shows the real Raman data for pure samples. Using the full spectrum with its many features was complicated to analyze. So, we chose to focus on two spectral windows which were particularly important for discriminating between the pure samples: the Raman shifts in the radial breathing mode (RBM) range (147–371 cm^−1^) and the G band range (1446–1645 cm^−1^). Figure 3a shows pure (6,5) nanotubes on the SiO_2_:Si substrates. The G^+^ band (1583 cm^−1^) maximum is about 150 counts/pixel over a 2 s integration time. However the RBM is barely visible, if at all, due to the substrate background. This is consistent with the fact that a weak RBM is expected for a high chiral angle SWCNT^23,24^, the fact that the G band is resonant for a wider range of excitation wavelengths than the RBM, and the fact that the (6,5) SWCNT RBM is further from resonance^5^. Figure 3b shows a (7,5) nanotube sample, with a much stronger G^+^ band (1584 cm^−1^, ~ 1300 counts/pixel at its peak or ~ 9× higher than the other species), mostly due to resonance, and a clear, strong RBM signal at 280 cm^−1^, about 450 counts/pixel above the background at its peak. The much weaker RBM at 261 cm^−1^ corresponds to a small amount of (*7*,*6*) contamination, which is also seen in photoluminescence (PL). (PL excitation maps of purified (*6*,*5*) and (*7*,*5*) materials are shown in the Supporting Material, Figs. S2, S3). There are some other small changes, particularly in the G band region. Figure 3c shows the signal from the bare substrate, which is very flat in the G band window, but has structure in the RBM region with a broad peak near 301 cm^−1^ which is, unfortunately, close to the expected position of the (*6*,*5*) RBM.

Figure 3d shows a series of experimental Raman maps for various dilutions with decreasing concentration of (*7*,*5*) SWCNT. Experimentally the laser is scanned stepwise across the surface of the sample in a grid of 30 by 30 pixels. The whole Raman spectra is measured at each point. The intensity plotted here comes from integrating over the (*7*,*5*) RBM peak area and subtracting a constant baseline, and so represents the abundance of (*7*,*5*) SWCNTs. The real maps, like the synthetic data maps of Fig. 1, decrease in intensity with dilution, except in the case of (vi). The slight increase brightness there is likely due to the G band being so weak that subtracting a flat background is no longer a good approximation. Figure 3d(iii) shows a clear hotspot, likely due to a cluster of SWCNT materials. Regions such as this which are obviously different are excluded from the subsequent analysis (see “Methods” section for details). It is noticeable that the real maps vary much more smoothly than the synthetic data. A more realistic simulation of our depositions, then, should include smaller fluctuations in numbers of SWCNTs from pixel-to-pixel.

We can situate the experimental data of Fig. 3 on the noise level graph from the synthetic data in Fig. 2e. The fluctuation in the background intensity for the Raman spectra is ~ 5 counts. The G band peak intensity at any given pixel at 3 × dilution is ~ 1300 counts, corresponding to ~ 4000 counts for an undiluted sample. Defining the signal-to-noise (S/N) as this ratio, we have S/N ~ 800 the inverse of which is ~ 10^–3^. At this noise level, the synthetic data suggests that abundances of ~ 10^6^ to ~ 10^7^ can be measured. This corresponds to dilution factors on 10^–3^ to 10^–2^ i.e. purities in the parts per thousand or parts per hundred level. As noted, our definition of noise level is quite harsh. Experimentally, we can increase the S/N by increasing the integration time, increasing the laser power, or improving the collection efficiency. Furthermore, the measurable purity level can only improve if more points are sampled in the spatial scan.

We applied the scikit-learn NMF algorithm to this data set. We found that the background signal was important to the output. So, a good experimental approach might be to use a background-free substrate. As shown in Fig. 3c the SiO_2_: Si substrate has a very nice, flat background in the G band region, however it is structured in the RBM region. We tried depositing the nanotubes on background-free aluminum substrates. However, in that case the experimental Raman signal was impractically weak, probably due to the electromagnetic node at the mirror-like substrate surface where the nanotubes were situated, and possibly also related to charge transfer between the nanotubes and the substrates. There are a number of substrate materials that may have been better, but there is always the question of obtaining good deposition on new types of substrates. So, we stayed with the SiO_2_: Si substrate as a compromise and instead tried data processing approaches to deal with any background.

We tried three main data processing approaches: (1) adding scans of bare substrates to the data set and allowing the NMF algorithm to “learn” the background as an additional factor (2) measuring the substrate signal, scaling its intensity to the background in the SWCNT spectra, and subtracting this to produce a background-free dataset (3) measuring the Raman spectra of pure (*6*,*5*) and (*7*,*5*) nanotubes and forcing the NMF algorithm to use these pure spectra as the components. All methods work to some degree, but we obtained much better matches to known dilution factors for (2) and (3), so we show those results below.

**Figure 4:**
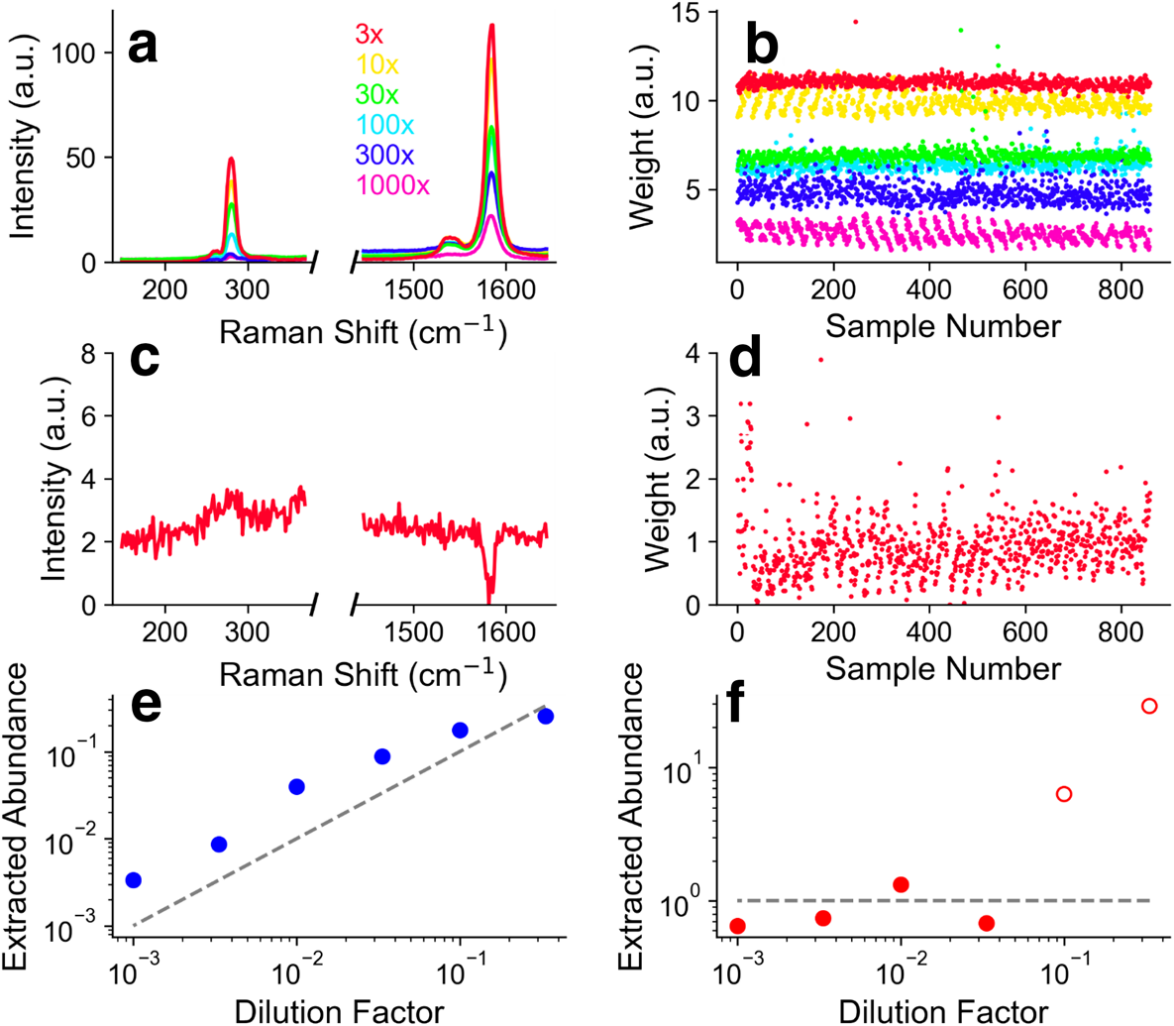
Analysis of real spectra with subtraction of substrate spectrum. (**a**) Extracted factor identified with minority (7,5) species for each dilution factor and (**b**) the corresponding weights. Colors in (**a,b**) represent different dilution factors. (**c**) An example of the extracted factor identified with the majority (6,5) species (**d**) the corresponding weights. (**e**) Extracted abundance of the minority (7,5) species at different dilution factors. The dashed line represents the expected true abundance. (**f**) Extracted abundance of the (6,5) species for different dilution factors of the (7,5) species. The open circles show poor agreement with experiment due to the (6,5) factor taking weight from the (7,5) tube.

Figure 4 shows the results of decomposing the real data with NMF where we have preprocessed the data by subtracting off the Si: SiO_2_ background (see details in “Methods” section). Figure 4a shows the extracted component corresponding to the minority factor, which is easily identified as the (*7*,*5*) SWCNT Raman spectrum. Figure 4b shows the corresponding weights. Figure 4c shows the other extracted component for one of the concentrations. It has no clear connection to the (*6*,*5*) spectrum. This is likely because the G band of the (*7*,*5*) species and (*6*,*5*) species overlap significantly, and the RBM of the (*6*,*5*) species is very weak, if present and overlaps with the substrate background. This situation could be improved experimentally by choosing another wavelength for excitation. For example we have done simultaneous two-wavelength excitation to excite two different resonances^25^, and recently developed continuous, “full spectrum” excitation to excite all resonances^26^. In any case, although hard to interpret, the weights Fig. 4d for this component are fairly consistent and stable.

By multiplying the components in Fig. 4a with the weights in Fig. 4b we get an experimentally derived abundance which can be compared to the dilution factor used to prepare the samples. The dotted line shows the abundance based on dilution factor, and the circles show the extracted abundance. Strictly speaking, the dilution factor is correct for the liquid dispersion. Although all samples were deposited in the same way, it is not necessarily true that the abundance on a deposited surface is directly proportional to the abundance in the solution used to prepare it. The adherence to the surface for different (*n*,*m*) could be different, also the degree to which the surface samples the bulk solution depends on mixing and fluid flow, which could be complicated. Furthermore, the different polymer content of the different dilutions and the rinsing of the substrate with solvent may cause changes in the abundance. Despite this potential complication, we find the extracted abundance scales rather well with the dilution factor.

There are notable periodic patterns in the extracted weights from the real data (Fig. 4b). This is an artifact of small, but systematic changes in the scattering signal as the laser spot is scanned across the sample. Non-uniform surface coverage could cause similar effects. However, we believe this stems from a small misalignment of the scan axis with the optical axis, leading to a small but systematic change in focusing, and so to the measured scattering intensities. Such effects are easy to miss by eye when capturing the data, but the spectral decomposition algorithm easily picks out such changes.

Figure 4f show the extracted abundance from the other component, presumably related to the majority (*6*,*5*) species. This is remarkably consistent for the low dilution factors. At the highest concentration of the minority (*7*,*5*) species it the abundance increases in an unphysical way (open circles). This is because, being more resonant, the (*7*,*5*) signal becomes very strong, and the algorithm mis-assigns weight from the (*7*,*5*) G band to the other component.

In the above, the NMF algorithm is free to seek out the components. But, it is possible to apply additional constraints. We applied the strictest possible constraints by forcing the algorithm to use the spectra of experimentally measured pure (*7*,*5*) and pure (*6*,*5*) SWCNT materials as components (see “Methods” section). It must be noted that forcing the NMF algorithm to use defined components for *all* components takes the “learning” out of the picture, and with a Euclidean distance metric this is essentially degenerate to a kind of direct classical least squares^2^ analysis, which is an older, and simpler method.

**Figure 5:**
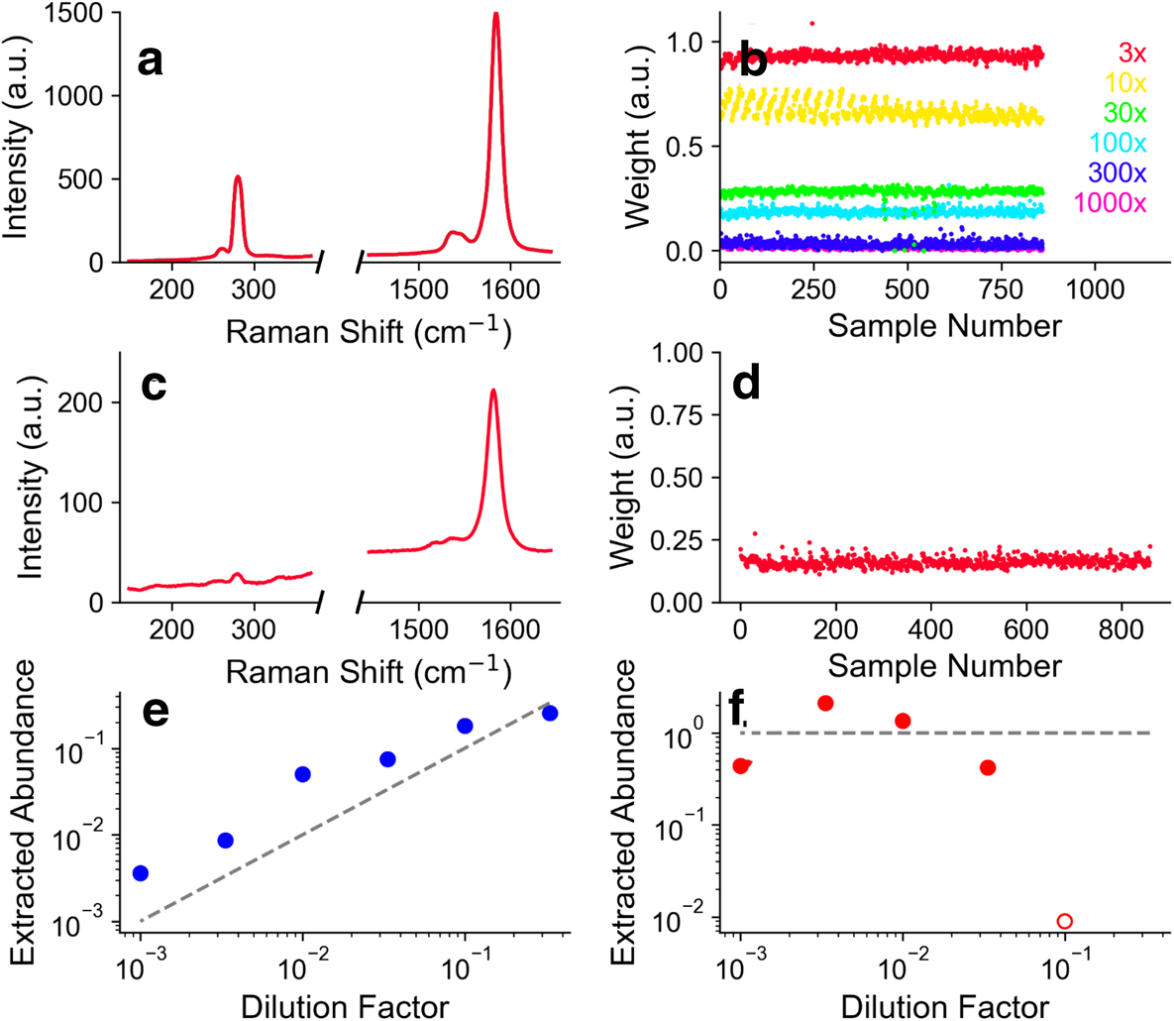
Analysis of real spectra by imposing custom components with subtraction of substrate spectrum. (**a**) Imposed (7,5) nanotube component, (**b**) weights extracted by the algorithm. Colors in (**a,b**) represent different dilution factors. (**c**) Imposed (6,5) nanotube component species, (**d**) weights extracted by the algorithm. (**e**) Extracted abundance of the minority (7,5) species at different dilution factors. The dashed line represents the expected abundance. (**f**) Extracted abundance of the (6,5) species for different dilution factors of the (7,5) species. The open circle show poor agreement with experiment due to the relative weakness of the (6,5) signal (see main text for details).

Figure 5a shows the imposed minority (*7*,*5*) species component, and Fig. 5b shows the extracted weights for each dilution factor. Figure 5c shows the imposed majority (*6*,*5*) species component, and (d) shows the corresponding weights, for one of the dilution factors as an example. The extracted abundance of the minority species is shown in Fig. 5e, with the dotted line showing the expected value based on dilution factor. The plot of the minority species abundance is remarkably similar to Fig. 4 which was “blind” and “learned” the (*7*,*5*) factor on its own.

The weights for the minority species are also fairly stable when the minority species is dilute. However, at high concentrations of the minority (*7*,*5*) SWCNT, the weight of the (*6*,*5*) species drops precipitously—basically to zero for the highest concentration of (*7*,*5*) SWCNT. This is because the NMF algorithm assigns most of the weigh to the stronger (*7*,*5*) species, paralleling the case for the analysis in Fig. 4.

## Conclusion

We have tested the ability of NMF, in its open-source python “scikit-learn” implementation, to determine the abundance of minority SWCNT species in mixtures deposited on substrates and probed by spatial mapping of Raman spectra. This decomposition approach efficiently evaluates high purity binary mixtures in an automated way. We have shown what minority species abundances are accessible at given noise levels. In both real and synthetic samples, we are able to measure minority species at dilution factors of 1000× with signal-to-noise levels that are easy to realize experimentally. Better results will be possible by adding more colors of excitation to track more chiralities on-resonance, and by focusing mainly on well differentiated peaks such as the RBM and G^−^ peaks, among others. Substrates which are background-free or featureless are preferred to prevent the algorithm from emphasizing spurious components which are not of interest. Just as forcing NMF to use known components was effective, we expect that adding extra—but less severe—constraints to the NMF search algorithm to prioritize spectral structures of interest will be worthwhile to push to higher purities, and for faster data acquisition and data processing.

## Methods

### Simulation details

The synthetic spectra for the (6,5) and (7,5) SWCNTs were created by summing up individual Lorentzian peaks for the RBM, D peak, G^−^ peak, G^+^ peak, and 2D peak. The wavenumber of the RBM peak was taken from Ref.^2^ and the G^−^ peak from Ref.^27^. We assigned both species G^+^, D and 2D peaks at 1582 cm^−1^, 1350 cm^−1^ and 2600 cm^−1^ respectively. For the RBM, D peak, G^−^ peak, G^+^ peak, and 2D peak of both species, the full width half maximums were set at 15, 45, 40, 30, and 30 cm^−1^ respectively. The intensities of the (6,5) peaks were set at 0.45, 0.15, 0.54, 1.25, and 0.25, and the (7,5) intensities were set at 0.37, 0.15, 0.45, 1.04, and 0.25. The intensity values were manually chosen to roughly resemble experimental data for nanotubes in general. These parameters are basically arbitrary. They should not be considered as a true representation of the actual parameters of real tubes under any specific conditions.

The SWNCT distribution on a sample substrate was simulated by producing an array of 900 rows of random floating point numbers, each number indicating how many simulated nanotubes were in a single pixel, with portions of complete nanotubes allowed. For a Raman image we used a 30 pixel × 30 pixel grid, with each pixel representing a 10 µm × 10 µm grid. To emulate a dense coverage of tubes with a fairly smooth spatial variation inspired by the variation that we regularly see on real samples, the random numbers followed a normal distribution with its mean being the total number of one billion (10^9^) tubes on the synthetic sample divided by the number of Raman mapping pixels (900). The standard deviation was set at 0.1 multiplied by the mean. The array of numbers was split into two sets for each chirality: the set for the (6,5) tubes had the same constant mean for each dilution factor, while the set for the (7,5) tubes had its mean decreased according to the current dilution factor. To generate the final spectra for each pixel, the base spectrum for each species was multiplied by its corresponding number of tubes in the current pixel, then the two multiplied spectra were added together. Flat noise was then added using a set of random values that had a normal distribution centered at a mean of 0 and its standard deviation being the noise level value. The simulation process was repeated with noise levels ranging from 10^–9^ to 1, and dilution factors ranging from 2^–30^ to 2^–1^ for each noise level. (Only the first six dilutions from 2^–1^ to 2^–6^ were plotted in Fig. 2a).

### NMF factorization details

Each prepared set of spectra data was inputted into the NMF algorithm of the scikit-learn decomposition library (version 0.24.02)^22^, which was set to decompose the spectra into two components (one for each chirality). The Coordinate Descent solver and the Nonnegative Double Singular Value Decomposition (NNDSVD) initialization were used to encourage sparseness in the outputted NMF factors^28,29^.

Multiple preprocessing steps were done on experimental Raman spectra data to prepare it for the NMF algorithm. The baseline of each CNT Raman spectra was reset to near zero using the average of the flat signal that came from a dark Raman scan. Outlier spectra that included abnormally high intensities or cosmic spikes were detected using z-scores. The z-score of each intensity value in the spectra was first calculated, then the spectra would be removed from the dataset if it contained a z-score larger than a manually chosen threshold. Typical chosen threshold values ranged from 8 to 12. Since the SiO_2_: Si substrate spectra was also present in the experimental spectra, two methods were used to help avoid having it interfere strongly with how the NMF algorithm detects the CNT spectra: The first method was to manually subtract the SiO_2_: Si background from dataset using the average of experimental SiO_2_:Si substrate scans. The second method was to pass the average spectra of pure (7,5), (6,5), and Si scans to the NMF algorithm as constant components, forcing the NMF algorithm to solve only for the weights of the three components. The pure (7,5) and (6,5) spectra were Si-background subtracted using the first method.

Extracted abundances were calculated by first summing up the weights of the NMF for each dilution factor. As discussed in the main text, NMF scales its factors in a non-unique way, so both the components and weights need to be scaled back relative to the original intensity values. This was done by multiplying by a factor equal to the ratio between the maximum NMF peak (the G^+^ peak) intensity and the maximum peak intensity of the original (6,5) and (7,5) spectra. For the experimental data, separate scans of only the pure (6,5) and the pure (7,5) species were used to get the maximum peak intensity of the original peak. For the synthetic data, the original base spectra were used.

### Nanotube source materials

Enriched, predominantly single chirality SWCNTs were prepared from unsorted SWCNT source material, CoMoCAT SG65i, purchased from Sigma-Aldrich (Cat# 773735). Two polyfluorenes were used: poly[(9,9-dioctylfluorenyl-2,7-diyl)-alt-co-(6,6′-{2,2′-bipyridine})] (PFO-BPy6,6′), purchased from American Dye Source Inc., and poly(9,9-di-n-octylfluorenyl-2,7-diyl) (PFO) was synthesized in our own laboratories.

The SWCNT source material (15.6 mg) was mixed with suitable polymer (15.6 mg) in 25 mL of toluene. For (6,5) SWCNT, the polymer was PFO-BPy6,6′ having molecular weight 34 kDa, and polydispersity 4.3, while for (7,5) SWCNT, the polymer was PFO having molecular weight 54 kDa and 2.4^30^. The mixture was probe-sonicated (Branson Sonifier 250) with a mini-tip of 3/16 inch at an output 30% and a duty cycle of 60% for 30 min, followed by centrifugation at 12,500 revolutions per minute (rpm) for 70 min (a relative centrifugal force of 18,700×*g*). The enrichment was repeated for multiple cycles to maximize the yield as demonstrated previously^31^. The UV–Vis–NIR absorption of the supernatant was measured by Agilent Cary5000 spectrometer in a quartz cuvette with an optical path of 4 mm.

### Sample preparation

SWCNT samples were prepared on silicon (100) substrates with 300 nm thermal oxide. Substrates were cleaned first by ultrasonication in acetone for 5 min, followed by 5 min of ultrasound in isopropyl alcohol (IPA). Substrates were blown dry using a stream of N_2_, then immediately placed in an ultraviolet/ozone cleaner for 30 min. Substrates were used within 20 min for the SWCNT deposition step. Suspensions of polymer wrapped semiconducting SWCNTs were used either directly from the undiluted above-prepared materials [(6,5) and (7,5) samples] or prepared as a geometric series of dilutions of the (7,5) stock liquid (minority species) in the (6,5) stock liquid (majority species). The SWCNT suspensions were subjected to ultrasound for 20 min prior to SWCNT deposition. For SWCNT deposition, a soaking method was used: Substrates were placed in a Petri dish, and then covered with a SWCNT suspension. The Petri dish was covered for 8 min to avoid evaporation of the solvent. After this time, the substrates were soaked for 5 min in toluene, then 5 min in IPA, and finally dried by a jet of N_2_. The samples were baked on a hot plate at 150 °C for 5 min.

### Spectroscopic measurements

The Raman spectra were collected by a home built Raman spectroscopic microscope used in Ref^32^, but with a fixed HeNe (632.8 nm) laser excitation. The laser was cleaned up by a laser line filter and directed at a microscope objective with a dichroic filter for 632.8 nm (Iridian Spectral Technologies). The microscope objective was a 50×, 0.42 numerical aperture, long working distance visible-near-infrared (Vis–NIR) objective. The spot size on the sample was ~ 3 µm in diameter. The Raman scattered light was collected by the objective, passed through the dichroic, and a linear polarizer so that the analyzed polarization was parallel to the excitation polarization (VV polarization). This was filtered by an edge filter and focused on a 10 µm wide slit by a 20 mm focal length lens (resulting in magnification of 5× rather than 50× for a 20 cm focal length lens. This matched the magnified spot well to the slit size. The signal was dispersed by a 0.328 m grating spectrometer (Andor Kymera 328i) using a 600 lines/mm grating blazed at 1 µm. Detection was with a long wavelength optimized charged-coupled device (CCD) camera (Andor iDus416). The incident laser power was 12 mW. This is very high power, but no degradation was seen in the Raman spectra over timescales of much longer than the integration time which was 2 s at each pixel, indicating that the power density was low enough. No confocal pinhole was used.

The sample was scanned spatially by custom python program driven absolute position encoded piezo-electric positioning stages. For spatial maps, a 30 × 30 grid (900 pixels) was raster scanned with lateral step sizes of 10 µm in both the *x* and *y* directions. The *z* (focus) axis was not adjusted during these scans.

The Raman shift was calibrated to an acetaminophen sample following standard protocols^33^. The Raman intensity was not corrected for instrument response.

## Supplementary Information

Supplementary Figures.

## Data Availability

Data are available from the authors upon reasonable request and with permission of the National Research Council Canada.

## References

[1] McCreery RL, Winefordner JD (2000). Raman spectroscopy for chemical analysis. Chemical Analysis: A Series of Monographs of Analytical Chemistry and Its Applications.

[2] Pelletier MJ (2003). Quantitative analysis using Raman spectrometry. Appl. Spectrosc..

[3] Jorio A, Saito R, Dresselhaus G, Dresselhaus MS (2011). Raman Spectroscopy in Graphene Related Systems.

[4] Lefebvre J, Finnie P, Fagan J, Zheng M, Hight Walker AR (2019). Metrological assessment of single-wall carbon nanotube materials by optical methods. Handb. Carbon Nanomater..

[5] Finnie P, Ding J, Li Z, Kingston CT (2014). Assessment of the metallicity of single-wall carbon nanotube ensembles at high purities. J. Phys. Chem. C.

[6] Li ZD (2015). Raman microscopy mapping for the purity assessment of chirality enriched carbon nanotube networks in thin-film transistors. Nano Res..

[7] Yang F, Wang M, Zhang D, Yang J, Zheng M, Li Y (2020). Chirality pure carbon nanotubes: Growth, sorting, and characterization. Chem. Rev..

[8] Jorio A, Saito R (2021). Raman spectroscopy for carbon nanotube applications. J. Appl. Phys..

[9] Gontijo R, Sáfar G, Righi A, Jain R, Strano M, Fantini C (2017). Quantifying (n, m) species in single-wall carbon nanotubes dispersions by combining Raman and optical absorption spectroscopies. Carbon.

[10] Tian Y, Jiang H, Laiho P, Kauppinen E (2018). Validity of measuring metallic and semiconducting single-walled carbon nanotube fractions by quantitative Raman spectroscopy. Anal. Chem..

[11] Castan A, Forel S, Fossard F, Defillet J, Ghedjatti A, Levshov D, Wenseleers W, Cambré S, Loiseau A (2021). Assessing the reliability of the Raman peak counting method for the characterization of SWCNT diameter distributions: a cross characterization with TEM. Carbon.

[12] Lee DD, Seung HS (1999). Learning the parts of objects by nonnegative matrix factorization. Nature.

[13] Szymańska-Chargot M, Pieczywek PM, Chylińska M, Zdunek A (2016). Hyperspectral image analysis of Raman maps of plant cell walls for blind spectra characterization by nonnegative matrix factorization algorithm. Chemometr. Intell. Lab. Syst..

[14] Deng X, Ali-Adeeb R, Andrews JL, Shreeves P, Lum JJ, Brolo A, Jirasek A (2020). Monitor ionizing radiation-induced cellular responses with Raman spectroscopy, non-negative matrix factorization, and non-negative least squares. Appl. Spectrosc..

[15] Milligan K, Deng X, Shreeves P, Ali-Adeeb R, Matthews Q, Brolo A, Lum JJ, Andrews JL, Jirasek A (2021). Raman spectroscopy and group and basis-restricted non negative matrix factorisation identifies radiation induced metabolic changes in human cancer cells. Sci. Rep..

[16] Deng X, Milligan K, Ali-Adeeb R, Shreeves P, Brolo A, Lum JJ, Andrews JL, Jirasek A (2021). Group and basis restricted non-negative matrix factorization and random forest for molecular histotype classification and Raman biomarker monitoring in breast cancer. Appl. Spectrosc..

[17] Liu X-Y, Guo S, Ramoji A, Bocklitz T, Rösch P, Popp J, Yu H-Q (2020). Spatiotemporal organization of biofilm matrix revealed by confocal Raman mapping integrated with non-negative matrix factorization analysis. Anal. Chem..

[18] Yakimov BP, Venets AV, Schleusener J, Fadeev VV, Lademann J, Shirshin EA, Darvin ME (2021). Blind source separation of molecular components of the human skin in vivo: Non-negative matrix factorization of Raman microspectroscopy data. Analyst.

[19] Albuquerque CDL, Poppi RJ (2015). Detection of malathion in food peels by surface-enhanced Raman imaging spectroscopy and multivariate curve resolution. Anal. Chim. Acta.

[20] Masia F, Glen A, Stephens P, Borri P, Langbein W (2013). Quantitative chemical imaging and unsupervised analysis using hyperspectral coherent anti-Stokes Raman scattering microscopy. Anal. Chem..

[21] Kunc J, Hu Y, Palmer J, Berger C, de Heer WA (2013). A method to extract pure Raman spectrum of epitaxial graphene on SiC. Appl. Phys. Lett..

[22] Pedregosa (2011). Scikit-learn: Machine learning in python. J. Mach. Learn. Res..

[23] Sato K, Saito R, Nugraha ART, Maruyama S (2010). Excitonic effects on radial breathing mode intensity of single wall carbon nanotubes. Chem. Phys. Lett..

[24] Piao Y, Simpson JR, Streit JK, Ao G, Zheng M, Fagan JA, Hight Walker AR (2016). Intensity ratio of resonant raman modes for (n, m) enriched semiconducting carbon nanotubes. ACS Nano.

[25] Li-Pook-Than A, Finnie P (2015). Observation of the metallic-type selective etching of single walled carbon nanotubes by real-time in situ two-laser Raman spectroscopy. Carbon.

[26] Finnie P, Ouyang J, Lefebvre J (2020). Full spectrum Raman excitation mapping spectroscopy. Sci. Rep..

[27] Telg H, Duque JG, Staiger M, Tu X, Hennrich F, Kappes MM, Zheng M, Maultzsch J, Thomsen C, Doorn SK (2012). Chiral Index Dependence of the G^+^ and G^–^ Raman modes in semiconducting carbon nanotubes. ACS Nano.

[28] Boutsidis C, Gallopoulos E (2008). SVD-based initialization: A head start for nonnegative matrix factorization. Pattern Recogn..

[29] Andrzej C, Anh-Huy PHAN (2009). Fast local algorithms for large scale nonnegative matrix and tensor factorizations. IEICE Trans. Fundam. Electron. Commun. Comput. Sci..

[30] Ozawa H, Ide N, Fujigaya T, Niidome Y, Nakashima N (2011). One-pot separation of highly enriched (6,5)-single-walled carbon nanotubes using a fluorene-based copolymer. Chem. Lett..

[31] Ouyang J, Ding J, Lefebvre J, Li Z, Guo C, Kell AJ, Malenfant PRL (2018). Sorting of semiconducting single-walled carbon nanotubes in polar solvents with an amphiphilic conjugated polymer provides general guidelines for enrichment. ACS Nano.

[32] Finnie P (2016). Tunable filter Raman spectroscopy of purified semiconducting and metallic carbon nanotubes. Nano Res..

[33] ASTM International (2014). Standard Guide for Raman Shift Standards for Spectrometer Calibration.

