# Supplementary material for: Nanotube abundance from non-negative matrix factorization of Raman spectra as an example of chemical purity from open source machine learning
Source: Sci Rep. 2022 Jul 8;12:11666. doi: 10.1038/s41598-022-15359-4 (PMC9270454; doi:10.1038/s41598-022-15359-4)
Supplement: Supplementary file 1 — Supplementary Figures. [file 41598_2022_15359_MOESM1_ESM.docx]

Supplementary Material for

Title: Nanotube abundances from non-negative matrix factorization of Raman spectra as an example of chemical purity from open source machine learning

Authors: Elijah Flores [1,2], Jianying Ouyang [1], François Lapointe [1], Paul Finnie [1,*]

Affiliations:

[1] National Research Council Canada, 1200 Montreal Road, Ottawa, Ontario, K1A 0R6

[2] University of Waterloo, 200 University Avenue West, Waterloo, ON, Canada, N2L 3G1

[*] corresponding author, email: Paul.Finnie@nrc-cnrc.gc.ca


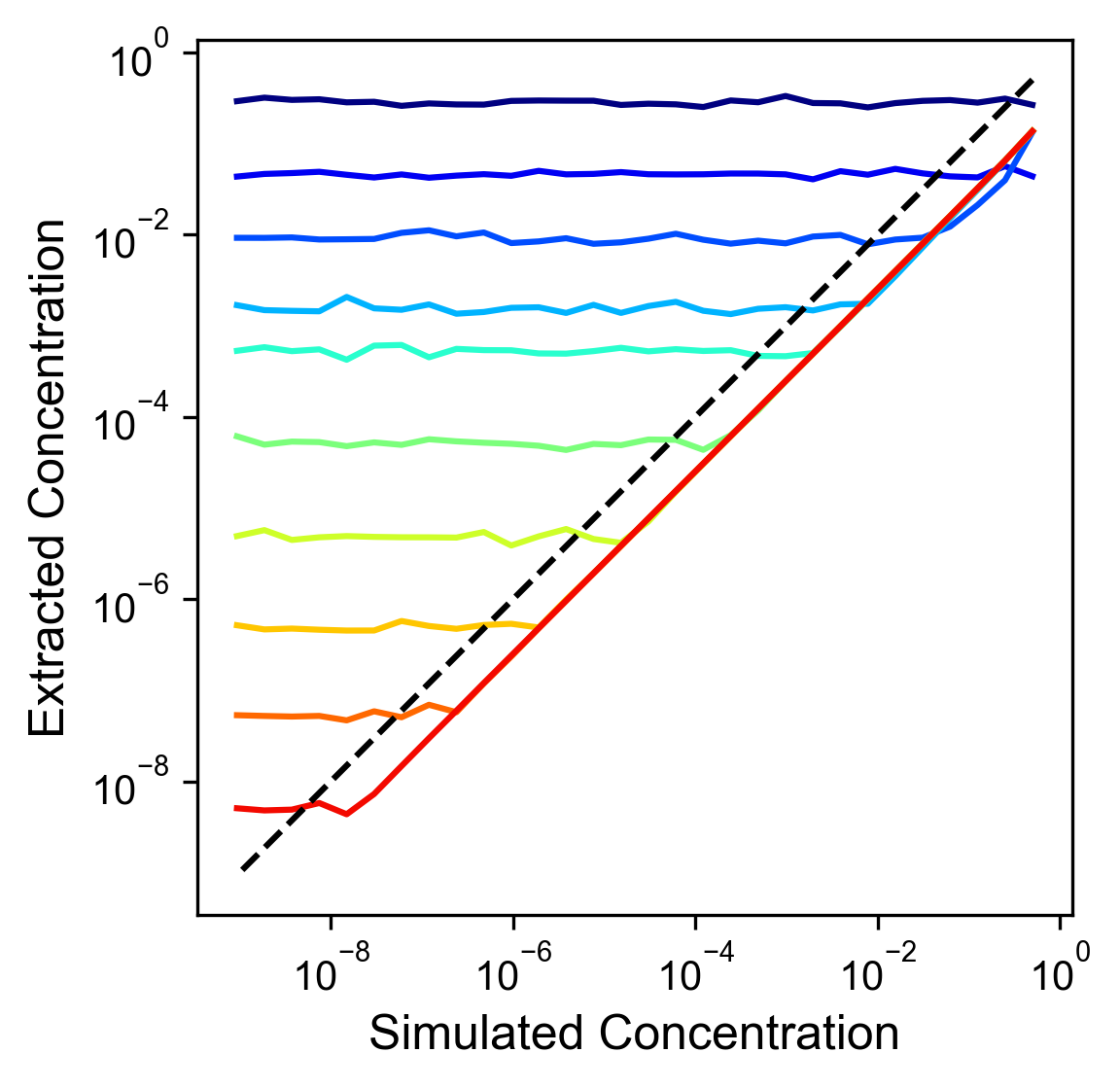


**Figure S1 Extracted Concentration vs. Simulated Concentration**

This is the same as Figure 2(e), but plotted in concentration. (See main text).


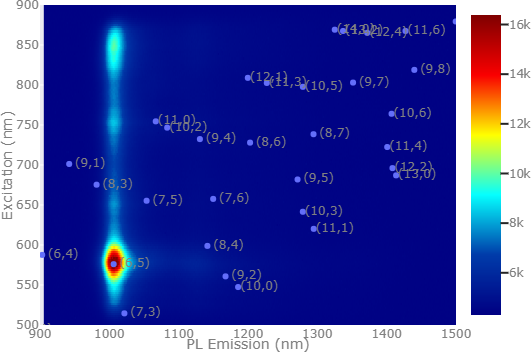


**Figure S2 Photoluminescence Excitation Map of (6,5) SWNCT Stock Liquid**


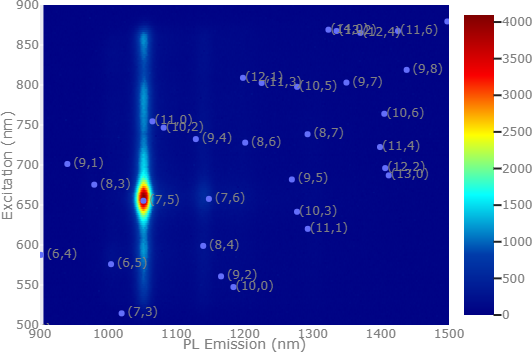


**Figure S2 Photoluminescence Excitation Map of (7,5) SWNCT Stock Liquid**

The photoluminescence excitation maps are taken with a real-time supercontinuum system after ref. [s1]. Positions of the *(n*,*m*) peaks are derived from ref [s2], allowing for a small dielectric shift due to the different environment, after ref. [s3]

[s1] J. Lefebvre, ACS Nano (2016) 9602

[s2] R. B. Weisman, S. M. Bachilo, Nano Letters(2003) 1235

[s3] J. Lefebvre, J. Fraser, Y. Homma, P. Finnie, Appl. Phys. A (2004) 1107
